# Evaluating the potential of chelation therapy to prevent and treat gadolinium deposition from MRI contrast agents

**DOI:** 10.1038/s41598-018-22511-6

**Published:** 2018-03-13

**Authors:** Julian A. Rees, Gauthier J.-P. Deblonde, Dahlia D. An, Camille Ansoborlo, Stacey S. Gauny, Rebecca J. Abergel

**Affiliations:** 1 Chemical Sciences Division, Lawrence Berkeley National Laboratory, Berkeley, CA 94720 USA; 2 Department of Nuclear Engineering, University of California, Berkeley, CA 94720 USA

## Abstract

Several MRI contrast agent clinical formulations are now known to leave deposits of the heavy metal gadolinium in the brain, bones, and other organs of patients. This persistent biological accumulation of gadolinium has been recently recognized as a deleterious outcome in patients administered Gd-based contrast agents (GBCAs) for MRI, prompting the European Medicines Agency to recommend discontinuing the use of over half of the GBCAs currently approved for clinical applications. To address this problem, we find that the orally-available metal decorporation agent 3,4,3-LI(1,2-HOPO) demonstrates superior efficacy at chelating and removing Gd from the body compared to diethylenetriaminepentaacetic acid, a ligand commonly used in the United States in the GBCA Gadopentetate (Magnevist). Using the radiotracer ^153^Gd to obtain precise biodistribution data, the results herein, supported by speciation simulations, suggest that the prophylactic or post-hoc therapeutic use of 3,4,3-LI(1,2-HOPO) may provide a means to mitigate Gd retention in patients requiring contrast-enhanced MRI.

## Introduction

**Figure 1:**
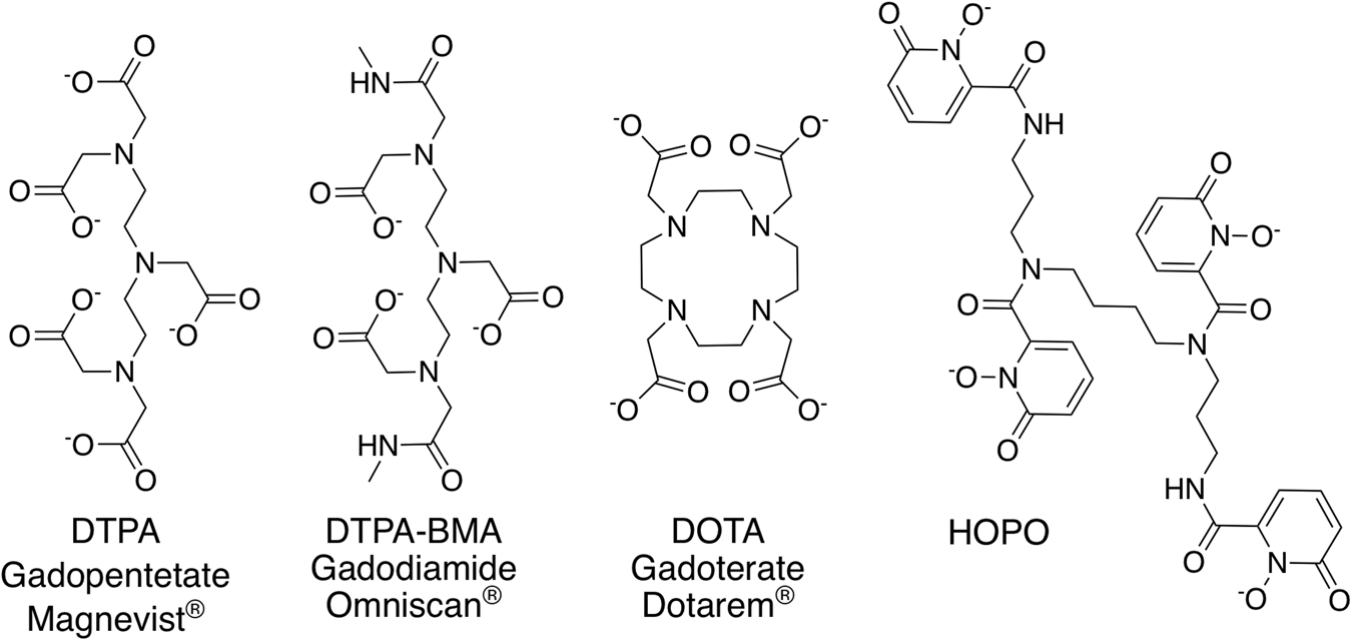
Structures of exemplary linear (DTPA, DTPA-BMA) or macrocyclic (DOTA) ligands used as GBCAs (generic and trade names are given for the corresponding Gd^3+^ complexes) and of the experimental chelator used in this study (HOPO).

The use of gadolinium-based contrast agents (GBCAs) for magnetic resonance imaging (MRI) has been ubiquitous in radiology for nearly three decades^1^. Recently however, it has come to light that the suboptimal biological stability of GBCAs can lead to accumulation of Gd in patients’ bone and brain tissue, as well as to kidney damage and associated systemic conditions due to compromised renal function^2–4^. These findings have prompted a substantial volume of new reports on the identification of residual Gd accumulation in contrast MRI patients, with the emerging consensus that GBCAs of the so-called “linear” type deposit more Gd into the body than their macrocyclic counterparts (Fig. 1). In one study, the linear contrast agent Gadodiamide (Omniscan), a derivative of the diethylenetriaminepentaacetic acid (DTPA) chelator currently in use as a metal decorporation agent^5^, was found to release 24% of its Gd^3+^ payload after only a single day in human serum; a number that increases to 64% in the presence of elevated phosphate levels^4^. In July 2017 the European Medicines Agency recommended suspending the intravenous use of Gadodiamide and Gadopentetate ([Gd(DTPA)]^2−^, Magnevist) in light of the available data, and in September 2017, the U.S. Food and Drug Administration’s Medical Imaging Drugs Advisory Committee recommended that prescribing information should include “a warning for retention for all GBCAs, with greater retention of all or some of the linear GBCAs compared to the macrocyclics in certain organs including the brain”.

While much of the literature has addressed both the cause and nature of metal accumulation *in vivo*^6^, little emphasis is placed on the prevention or remediation of Gd deposition. In this study, the chelating agent 3,4,3-LI(1,2-HOPO), or HOPO, is evaluated relative to DTPA as a preventive treatment for Gd accumulation (Fig. 1).

HOPO is an octadentate hydroxypyridinone ligand that is among the most competent chelating agents for heavy f-block metals (log *K*_ML_ at pH 7.4 for Gd of 20.5(1))^7^. It is orally available, non-toxic at therapeutic dosages, and is extremely selective for actinide and lanthanide ions over biologically-relevant metal ions such as iron, potassium, and calcium^8^. These are highly advantageous properties for a therapeutic chelator, and thus HOPO has been approved for a first-in-human Phase 1 safety trial as an actinide decorporation agent^9^. This led us to surmise that HOPO could provide an effective treatment for preventing and remediating the biological accumulation of Gd that has been observed in numerous recent studies. To evaluate this hypothesis, the respective efficacies of HOPO and DTPA as decorporation agents for the radiotracer ^153^Gd were probed in a murine model^8,10^.

## Results

### Bodily ^153^Gd Accumulation

**Figure 2:**
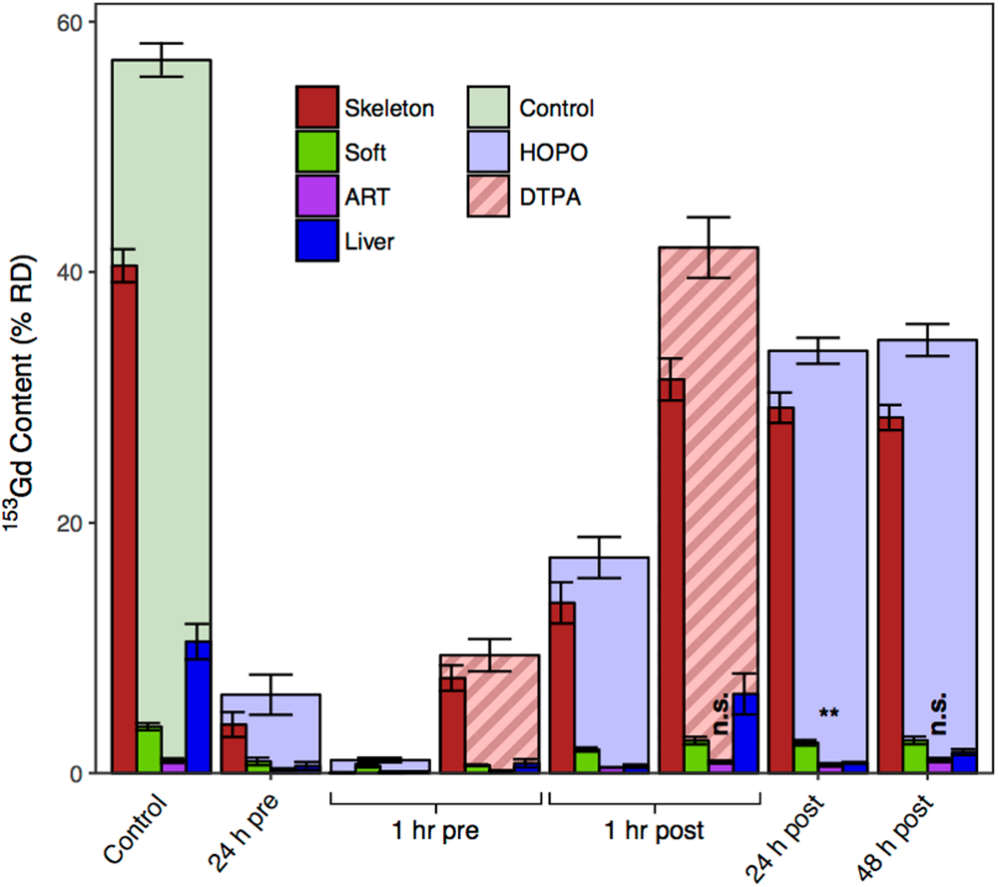
Total murine body content and distribution of ^153^Gd four days following contamination via intravenous injection into a warmed lateral tail vein, reported as % RD. Treatments were administered at a dose of 100 *μ*mol/kg via ip injection at times varying from 24 h pre- to 48 h post-Gd injection. Analyzed organ systems that comprise at least 1% RD are shown. Unless otherwise indicated, the results of one-way ANOVA (Dunnett’s method) yielded *p*-values < 0.001 (n.s., not significant, **p < 0.01).

Intraperitoneal (ip) administration of both HOPO and DTPA was found to promote the clearance of ^153^Gd, and for both chelators, the extent of clearance varies with administration time relative to ^153^Gd contamination. Figure 2 shows the total percentage of the Gd dose recovered from the subject animals for each treatment group (%RD), as well as the distribution of ^153^Gd found in selected organ systems. In the absence of treatment nearly 60% of ^153^Gd was retained in the body after 4 days; primarily from the skeleton (41% RD), soft and abdominal tissues (4% RD), and excretory organs (liver 11% RD, kidneys 1% RD). These data reflect the established propensity of heavy metals to accumulate in the skeleton, and it is noted that this biodistribution, particularly the elevated liver accumulation of Gd compared to other non-skeletal organ systems, is consistent with previous reports^11,12^. While all treatment groups in the present study show an increase in cleared ^153^Gd, three are notable in their ability to reduce the amount of retained metal to under 10% RD. Treatment with DTPA 1 h prior to contamination lowered the amount of bodily^153^ Gd to 9.4 ± 1.3% RD, while administration of HOPO either 24 h or 1 h prior to contamination reduced the amount of retained metal to 6.3 ± 1.6% RD and 1.04 ± 0.18% RD, respectively. The latter treatment regimen corresponds to more than a 50-fold decrease in bodily ^153^Gd content compared to the control group, but more importantly a roughly 9-fold decrease relative to DTPA, one of the linear GBCAs currently approved for clinical use in the United States.

**Figure 3:**
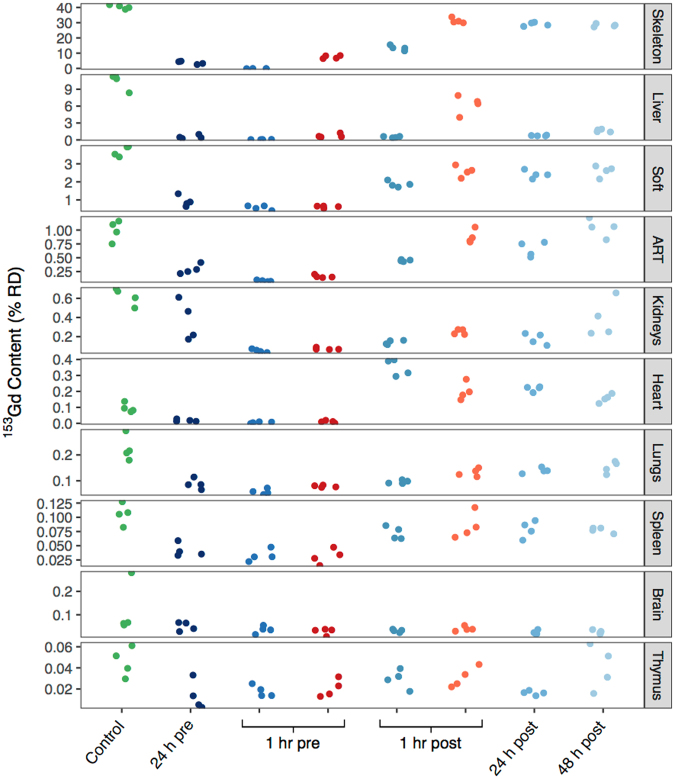
Murine organ content of ^153^Gd four days following contamination, reported as %RD. Data points correspond to the values obtained for each mouse in a group (*n* = 4). The data points on the right side of the 1 hr pre and 1 hr post groups (red and orange) received treatment with DTPA, and all other non-control groups received HOPO. Organs are ranked in order of contribution to the total RD.

Of particular interest are the organs that exhibit substantial levels of Gd in this model, as well as those that show lasting Gd accumulation in human patients with potentially negative health outcomes: skeleton, liver, kidneys, and brain. Figures 2 and 3 show that for all treatment groups except a 1 h pre-treatment with HOPO, skeletal ^153^Gd makes up the majority of the recovered bodily content. For that particular treatment however, there is complete prevention of Gd accumulation in the skeleton. As previously mentioned approximately 11% RD was recovered from the liver in the control group (Tables S1, 2, Fig. 3). Interestingly, all of the treatments succeeded in reducing this burden to <2% RD except 1 h post-contamination DTPA, where 6.3 ± 1.6% RD was found in the liver (2). The substantially lower liver content of the 1 h pre-contamination DTPA treatment (0.78 ± 0.33% RD) suggests that while DTPA can effectively chelate Gd in other organ systems and prevent its accumulation in liver tissue, either it cannot effectively compete for Gd binding with hepatic proteins or small molecules, or it simply does not reach appreciable concentrations in liver tissue. In contrast, administration of HOPO even 48 h following contamination reduces the ^153^Gd liver content to 1.68 ± 0.24% RD, suggesting that HOPO is capable of diffusing to the highly vascularized liver tissue and also exhibits a higher affinity for Gd than do endogenous biomolecules.

There is now a substantial body of literature implicating Gd from GBCAs in the onset of nephrogenic systemic fibrosis (NSF) in patients with suppressed renal function^13,14^. Thus, the accumulation of Gd in kidneys is of importance. Nephrogenic ^153^Gd accumulation in the control group accounts for 0.62 ± 0.09% RD, and all treatment regimens were able to promote a reduction in ^153^Gd content. A 1 h pre-contamination treatment of either HOPO or DTPA showed the most substantial effect, resulting in statistically-equivalent burdens of 0.06 ± 0.02% and 0.08 ± 0.01% RD, respectively. For both chelators, delayed administration increases the ^153^Gd kidney concentration, suggesting that their ability to recover ^153^Gd is impaired over time. Given that the chelators’ pharmacokinetic profiles should not be impacted by the administration time relative to contamination, the data may indicate that progressively, kidney physiology could promote formation of Gd species that are more resistant to chelation by DTPA or HOPO.

Recent studies have also highlighted the accumulation of Gd in the brain tissue of otherwise healthy patients, and the degree to which linear and macrocyclic GBCAs lead to such deposition remains an area of active debate^3,15–18^. Accordingly, the accumulation of ^153^Gd in the brain tissue was examined in greater detail. In the control group, an outlying data point (Fig. 3) was detected and removed (*Q*-value >0.95), to give a corrected concentration of 0.063 ± 0.003% RD. This value is second only to the thymus for the *least* amount of accumulated ^153^Gd, and there is no statistical difference between groups, seemingly contrary to many of the studies referenced previously. However we note that as the timescale of intra-cranial Gd deposition is unclear, the experiment duration (4 d) was possibly too short to observe appreciable accumulation in the brain. Additionally, the physiological Gd concentration in the present study is at radiotracer levels; roughly 100 million times lower than therapeutic concentrations for contrast MRI. This may simply be too low to observe intracranial accumulation if there is a threshold concentration for Gd transiting the blood-brain barrier.

### ^153^Gd in Excreta

**Figure 4:**
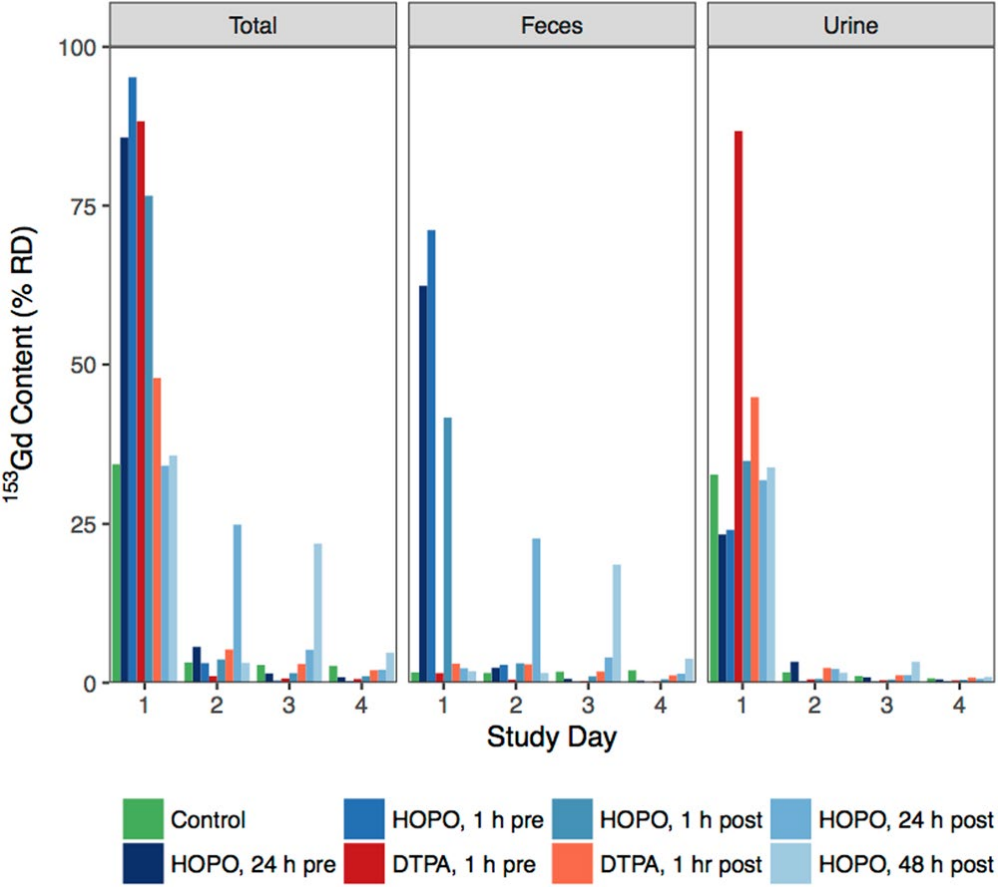
Excreted ^153^Gd by study day and excretion route, reported as %RD. Treatments were administered at a dose of 100 *μ*mol/kg via ip injection at times varying from 24 h pre- to 48 h post-Gd injection. Treatment groups administered HOPO show significant enhancement of fecal excretion relative to both control and groups receiving DTPA.

In addition to bodily accumulation, the excreta were collected daily and assayed for ^153^Gd content. Figure 4 shows the ^153^Gd recovered from the excreta of all study groups, as well as the relative amounts found in the urine and feces. The excreta of the control group contained 34.4% RD on day 1, with a roughly 95% urine and 5% feces ratio. On all subsequent days an additional ≈3% was found in the total excreta. Interestingly, while on day 2 an equal amount of ^153^Gd was found in the urine and feces, on days 3 and 4 the ratio of excreted Gd shifted towards increased concentration in the feces, indicating that while the urinary metabolism is responsible for the initial clearance of Gd, over time the remaining metal is cleared through solid waste. For the treatment groups, the cumulative total excreta on day 4 are, as expected, the inverse of the total bodily accumulations (Figure S1). The most dramatic difference between treatment groups is that upon treatment with HOPO, a substantial increase in fecal clearance is observed, whereas DTPA promotes clearance through the urinary pathway only. This is most clearly observed in the groups receiving HOPO 24 and 48 h post-contamination; those treatment groups are statistically identical to the control until treatment, whereupon a significant increase in fecal excretion is found. It is noted that this is consistent with the observed capacity of HOPO to reduce liver concentrations of ^153^Gd when administered 48 h post-contamination. In contrast, DTPA is cleared via the renal pathway, and even a 1 h post-contamination administration leaves substantial Gd accumulation in the liver tissue.

## Discussion

### Biodistribution and Decorporation

The data presented here illustrate the fundamental propensity of Gd^3+^ to accumulate in various organ systems, and provide insight into the strengths and weaknesses of DTPA as a prototypical linear GBCA ligand. The primary purpose of this study was to determine the biodistribution of Gd and understand the changes engendered by two different chelating agents, and to ultimately inform the design or selection of chelators that are more ideally compatible with the biochemistry of Gd. While it is clearly of interest to consider the implications of these findings in light of present concerns regarding the widespread use of GBCAs, we caution that a few key differences preclude direct comparison of these data to post-hoc studies of clinical subjects. First, the concentration of the ^153^Gd radiotracer (≈0.67 pmol/kg) was tailored to provide accurate dosimetry without unnecessary radiation exposure to either the subject animals or the researchers, and as mentioned previously the concentrations of Gd are lower than clinical doses (0.1 mmol/kg) by roughly seven orders of magnitude, adjusting for the human-equivalent dose^19,20^. Additionally, clinical formulations of Gd-DTPA only contain a 0.1% excess of DTPA^4^, however in the present study the chelator concentrations were 100 *μ*mol/kg, a >10 million-fold excess. A higher level of Gd deposition can therefore be expected in the case of an actual Gd-DTPA MRI formulation^20^.

Despite these deviations from clinically-relevant conditions, the varying effects of the chelation treatments on ^153^Gd biodistribution give rise to important conclusions regarding the biochemistry of Gd in various organ systems. Deposition in the skeleton is the ultimate fate of a substantial fraction of the retained contaminant. However, consistent with previous studies on the decorporation of other f-elements such as ^238^Pu^4+^ ^21,22^, ^241^Am^3+^ ^19^, or ^152^Eu^3+^ ^7^, HOPO is more effective than DTPA at reducing the overall skeletal content of the heavy metal, even when administered up to 48 h post-contamination. In comparison, administration of DTPA only 1 h post-contamination cannot prevent Gd accumulation in the skeleton as efficiently. It is noted that our previous studies examining the chelation of ^238^Pu by HOPO determined that administration even 7 d post-contamination was as efficacious as administration 1 d post-contamination; were the present study extended further, we posit that similar behavior could be anticipated. It is also probable that repeated administration of HOPO could further improve the clearance of Gd from the body.

Synthesis of the concentration data from the liver, kidneys, and excreta derives a more comprehensive picture of the differences in pharmacokinetics between DTPA and HOPO. The inability of DTPA to retroactively clear Gd from the liver, even at 1 h post-contamination, suggests that either the biochemistry of liver tissue suppresses complexation of Gd, or that DTPA is rapidly metabolized via the kidneys and has negligible circulation through the liver. The almost exclusive elimination of Gd in the urine following treatment with DTPA indicates that the latter is, in our opinion, more likely. In contrast, HOPO effectively removes ^153^Gd from the liver even if administered 48 h post-contamination. Coupled with the sizable increase in fecal excretion upon treatment with HOPO, as well as the negligible fecal clearance of the control group, the data present a compelling case that the Gd-HOPO complex is metabolized through the liver and cleared as solid waste. While the use of linear GBCAs in patients with advanced kidney disease has been largely discontinued, the genesis of Gd-induced nephrotoxicity and the potential implications for otherwise healthy patients have not been established. Thus, it would seem that a decorporation strategy that does *not* utilize the renal excretion pathway could be advantageous.

Finally, in the present study, treatment with a chelating agent had statistically no influence on the extent of ^153^Gd recovered from the brain tissue; however as discussed previously it is highly possible that either the timescale or the concentration regime of this experiment precluded the observation of any treatment group-dependent trends. Intra-cranial Gd deposition has received substantial attention in the recent literature, with mounting evidence that even in patients with uncompromised renal function, Gd will accumulate in various regions of the brain^1,16,18,23^. Additionally, efforts to characterize the speciation of Gd in both brain and other tissues have identified insoluble Gd-containing deposits high in phosphate and calcium, strongly suggesting that Gd is no longer associated with the intended chelating agent^6,24^. As such, it would seem imperative that more effective chelators be employed. While the present data reveal no specific trends regarding Gd deposition in brain tissue, the overall decorporation efficacies indicate that HOPO is a far better chelator of Gd compared to DTPA, particularly if the present data are indicative of delayed intracranial Gd accumulation; in this case HOPO would be expected to substantially mitigate this problem.

### Gd Speciation in Physiological Conditions

In the present context, the effectiveness of a chelator is determined by its ability to maintain coordination *in vivo*, which is of importance for preventing the precipitation of Gd deposits in the body. To properly address this question the speciation of Gd must be considered in the presence of not only the chelator, but also additional biological anions capable of coordinating the metal. The *in vivo* chemistry of exogenous metals such as Gd is far too complex to be modeled completely, as it would require knowledge of the thermodynamic and kinetic properties of all potential ligands (small inorganic and organic molecules, transport proteins, etc.). Ideally, the effect of the ionic strength, temperature, viscosity, local concentration and interference with essential cations such as Fe^3+^, Ca^2+^, and Zn^2+^ would also be taken into account, but these necessary data are not known for many of the chelators utilized in GBCAs. We have therefore performed speciation simulations for the most common Gd^3+^-MRI chelator systems, utilizing published thermodynamic parameters and accounting for the presence of the most abundant and relevant natural chelators: phosphates, carbonates, hydroxides, oxalates, lactates, and citrates, as well as the synthetic chelators themselves (Table S3).

**Figure 5:**
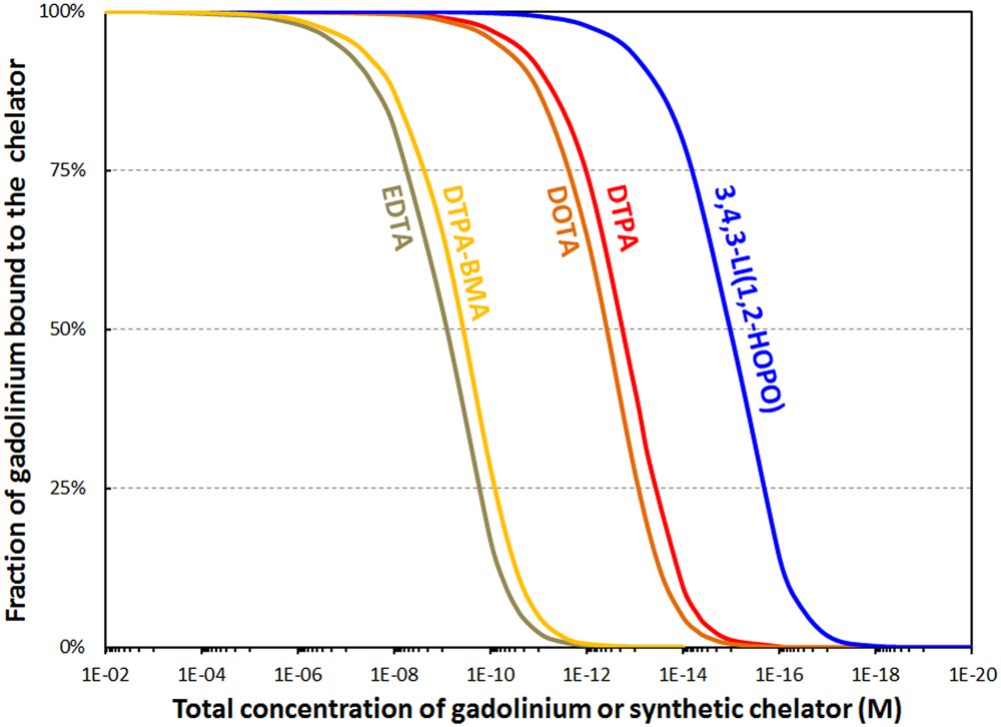
Calculated percentage of gadolinium bound to various chelators relevant for MRI as a function of dilution and under biological conditions. Total concentrations of phosphates (1.1 mM), carbonates (25 mM), oxalates (9.2 μM), lactates (1.5 mM), and citrates (160 μM) held constant to match physiological conditions. Ratio Gd/synthetic chelator = 1.0 mol/mol. pH = 7.4. See Table S3 for stability constants used in these speciation simulations.

Parameterization of metal complex stability to account for chelators of different nature, acidity, and denticity in a biological environment is a nontrivial problem. To enable a direct comparison of common exogenous chelators utilized for Gd and other metals *in vivo*, the simulations performed here consisted of lowering the concentration of Gd and the synthetic chelator at constant pH and concentration of endogenous ligands. This aims to mimic the progressive dilution or clearance of the GBCA from the blood stream, and as the concentrations decrease at static conditions of the other ligands, ultimately the chelator is unable to compete with the biological molecules and Gd is released. The concentration at which this is predicted to occur allows for the evaluation of the relative stability of the synthetic chelators post-treatment. Figure 5 shows the results of the simulations for the Gd-chelator complexes of ethylenediaminetetraacetate (EDTA), DTPA-BMA, DTPA, DOTA, and HOPO (Fig. 1), in the presence of competing biological ligands and at physiological pH. We chose DTPA-BMA, DTPA, and DOTA as models for speciation calculations in part due to the availability of thermodynamic parameters for these particular ligands, but mostly because all other currently used GBCAs are derived from these molecular structures.

EDTA has been included because it is available and commonly used for chelation therapy of lead, another heavy metal. Figure 5 suggests that HOPO outperforms all challenge chelators by at least two orders of magnitude. In line with the *in vivo* studies with Gd ions, the binding ability of the synthetic chelators extrapolated from the speciation simulation is as follows: EDTA = DTPA-BMA < DTPA = DOTA < HOPO. The simulation does not however account for differences in kinetic inertness between chelators; while the macrocyclic DOTA chelator for example has a lower stability constant than DTPA, the activation energy for demetallation is substantially greater, and DOTA has thus emerged as a more stable chelator than DTPA *in vivo*^4,25,26^. A few additional points from this simulation should be noted. First, while numerical values are provided on the x-axis, they should be taken as relative stabilities rather than absolute: Other endogenous challengers (such as metal-binding proteins and other metal ions)^27^ are present in actual biological media in addition to those taken into account in the present speciation studies. Therefore the thresholds at which the synthetic chelators release Gd are expected to be higher in actuality, but this offset due to other natural ligands is expected to be very similar.

**Figure 6:**
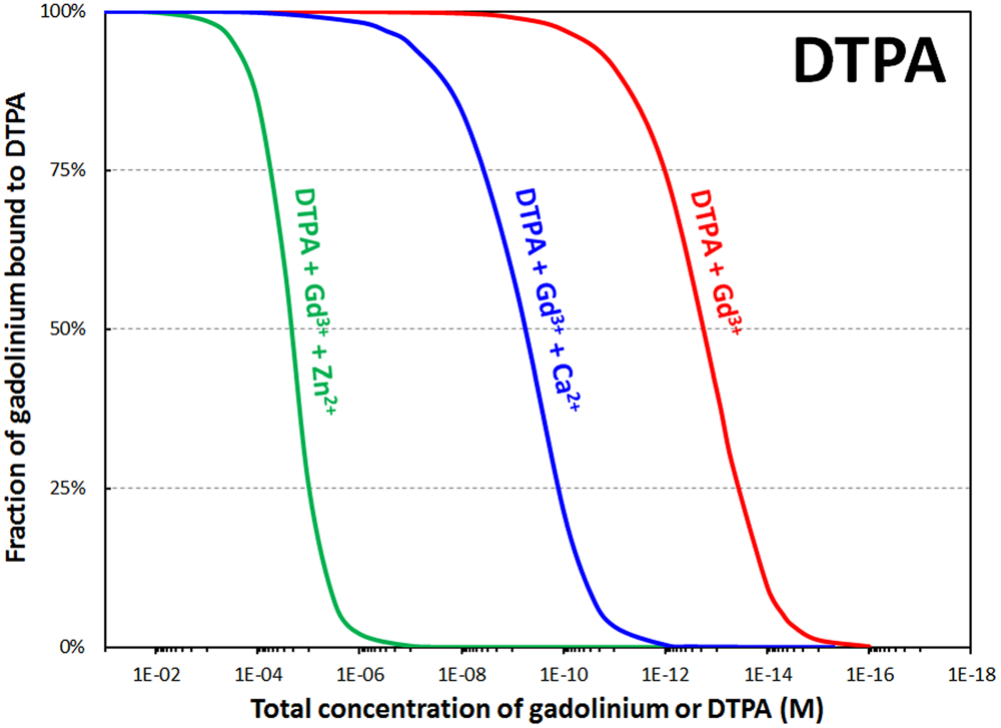
Calculated percentage of gadolinium bound to DTPA as a function of dilution in the presence of biological chelators, and with inclusion of physiological concentrations of calcium (1.1 mM) or zinc (15 *μ*M). Total concentrations of phosphates (1.1 mM), carbonates (25 mM), oxalates (9.2 μM), lactates (1.5 mM), and citrates (160 μM) held constant to match physiological conditions. Ratio Gd/synthetic chelator = 1.0 mol/mol. pH = 7.4. See Table S3 for stability constants used in these speciation simulations.

Once the Gd is released, an equilibrium is predicted between citrate, carbonate, and phosphate (Figs S3–4), however precipitation of Gd phosphate, the least soluble species, depletes that term of the equilibrium expression. In an abundance of phosphate, the equilibrium is expected to shift, inducing continued precipitation and formation of Gd phosphate deposits, as has been reported in the tissues of patients receiving GBCAs^6,24^. Further, speciation was also calculated for the Gd-DTPA and Gd-DTPA-BMA complexes in the presence of Ca^2+^ and Zn^2+^ ions, since the stability constants of the Ca-DTPA, Ca-DTPA-BMA, Zn-DTPA, Zn-DTPA-BMA, and the Ca^2+^ and Zn^2+^ complexes of the relevant endogenous ligands are known (Table S3). The *in vivo* exchange of metal ions, commonly referred to in the literature as transmetallation, is a compelling theory that rationalizes the release of Gd^3+^ in the body, and our results show an important Gd^3+^-Zn^2+^ transmetallation effect for both linear chelators. Ca^2+^ is a weaker competitor for GBCAs than Zn^2+^ but both endogenous metals can displace Gd^3+^ from the pharmaceutical complexes below a certain concentration (Fig. 6, Figure S5).

These simulations provide excellent rationale for the elevated levels of urinary Zn (zincuria) that have been observed in both human and animal studies^13^, given that our findings indicate urinary clearance of the M^*n*+^-DTPA complexes^7,19,21,22^. It then seems likely that Zn transmetallation of GBCAs in the kidneys could be implicated as an underlying cause of NSF.

These speciation simulations were based on published thermodynamic parameters, and are to the best of our knowledge wholly supported by the available clinical data. In theory, the observed *in vivo* behavior of these linear GBCAs, both in terms of phosphate precipitation and Zn transmetallation, could have been predicted from such simulations prior to their clinical use. Consideration of biologically-relevant metals as coordination complexes, both exogenous and otherwise, can provide rational solutions to problems such as release of Gd *in vivo*, utilizing existing knowledge of the coordination preferences of alkali earth, transition, and heavy metals. DTPA and most other GBCA chelators are promiscuous in their chelation of metal ions, and the adverse chelation of Zn^2+^ and other vital biological cations such as Ca^2+^, Mg^2+^, and Fe^3+^ is a key reason for the inherent toxicity of DTPA and other chelators^28^.

In stark contrast, the HOPO chelator is, by design, highly selective for lanthanide and actinide ions. This octadentate ligand binds metal ions through the eight oxygen donor atoms of its four bidentate binding units, and is ideally suited to the larger f-block cations, while disfavoring complexation of transition metals and Group I or II ions ubiquitous in biology. In fact, the maximum tolerated dose of HOPO in rats was determined to be in excess of 100 *μ*mol kg^−1^ d^−1^ over 28 days of daily oral administration^9,19,22^. The higher acidity of HOPO (highest p *K*_a_ = 6.6) compared to DTPA (10.4) or DOTA (11.2) makes it more resistant to intrinsic competition between ligand protonation and metal binding at low concentrations. Due to its high denticity and steric bulk, we anticipate Gd-HOPO to be an ineffective MRI contrast agent, since all coordination sites of Gd^3+^ should be occupied and no water molecules are expected within the first coordination sphere, consistent with findings from time-resolved fluorescence measurements on the corresponding Eu^3+^ complex^7^. However, this same property makes it an excellent Gd-decorporation molecule compared to the GBCA chelators examined herein. Thus, one could envision a scenario where a currently-approved GBCA is administered, the MRI is performed, and a subsequent regimen of HOPO is prescribed to chelate and remove any Gd^3+^ that dissociates from the GBCA^29,30^.

In light of current data, the oral availability, safety, and selectivity for f-block elements make the metallobiochemistry of HOPO ideally suited to address the issue of Gd accumulation in patients receiving GBCAs. Subsequent experiments will address key outstanding questions; namely, how do the differences in chelator and Gd^3+^ concentration and stoichimetry impact biodistribution, and does the co- or pre-administration of HOPO adversely impact the ability of GBCAs to provide the *t*_1_ relaxation enhancement needed for contrast MRI. Finally, we plan to investigate if HOPO can provide improved clearance of Gd^3+^ from mice following dosing with clinically-relevant concentrations of linear and macrocyclic GBCAs. These studies will employ a wider range of GBCAs and treatment conditions, as well as therapeutic levels of Gd spiked with the ^153^Gd radiotracer for concurrent dosimetry. Considering the many patients who have undergone contrast MRIs and may have bodily Gd accumulation, the present data suggest that the HOPO chelator could provide improved retroactive chelation treatment compared to other ligands currently used for such applications. Previous work has shown that HOPO will circulate to the brain and bones^31^, and also that delayed treatment with HOPO is effective at removing ^238^Pu^4+^ from mice^21^. We note that the timescale of contamination relative to treatment, as well as the chemical speciation of deposited Gd, are additional factors to be explored when evaluating HOPO as a post-hoc Gd remediation therapy.

## Conclusions

From the perspective of coordination chemistry, the design of a successful agent for contrast MRI is somewhat paradoxical. The ligand must have sufficient affinity and selectivity for Gd so as to be stable *in vivo*, either from competition for Gd by endogenous ligands or transmetallation of the chelator by bioavailable metals; however, without at least one open coordination site for water to bind, *t*_1_ relaxation enhancement is quenched and the complex ceases to perform as a contrast agent. Thus, many of the best ligands for chelating Gd, including HOPO, may be unsuitable as contrast agents because the chelator occupies all available coordination sites. Recognizing the value of the clinical data afforded by contrast-enhanced MRI, such insight inspires a critical evaluation of the current strategies employed for GBCAs, and in particular raises the question of whether a single-chelator approach is in fact the safest and most effective means to provide MRI contrast. We eagerly anticipate the creative consideration of the points raised herein, as well as the properties of f-block-specific chelators such as HOPO, in the rapidly-expanding body of literature in this field.

## Methods

### Metal and Ligand Solutions

A stock solution of ^153^Gd-chloride in 1 M HCl was purchased from Eckert and Ziegler Isotope Products (Valencia, CA, USA). Contamination doses consisted of 0.2 mL aliquots of solutions containing 0.925 kBq (6.94 pg) of ^153^Gd in 0.008 M sodium citrate and 0.14 M NaCl, pH 4. 3,4,3-LI(1,2-HOPO) was prepared by Ash Stevens, Inc. (Detroit MI, USA), as described previously^9^. DTPA was obtained from Sigma-Aldrich (St. Louis, MO, USA) and was formulated as Ca-DTPA using CaCO_3_ and NaOH, similar to the formerly available drug product commercialized by Hameln Pharmaceuticals GmbH (Hameln, Germany). Ligand solutions were prepared such that the dosage of 100 *μ*mol/kg was contained in 0.5 mL of 0.14 M NaCl, with the pH adjusted to 7.4–8.4 with 1 N NaOH. All solutions were filter-sterilized (0.22 *μ*m) prior to administration.

### Animals and General Procedures

All procedures and protocols used in the described *in vivo* studies were reviewed and approved by the Institutional Animal Care and Use Committee of Lawrence Berkeley National Laboratory, and were performed in AAALAC-accredited facilities according to prescribed guidelines and regulations. The animals were young adult (102 days old) female (31.7 ± 2.1 g) Swiss-Webster mice (Simonsen Laboratories, Gilroy, CA, USA). Gross body and tissue compositions, plasma, extracellular fluid, and red cell volumes of the whole body, major tissues and organs of these mice have been determined previously^32^. Mice were kept under a 12 h light cycle with controlled temperature (18–22 °C) and relative humidity (30–70%), and were given water and food *ad libitum*. Each group was housed together in a plastic stock cage lined with a 0.5 cm layer of highly absorbent low-ash pelleted cellulose bedding (ALPHA-dri) for separation of urine and feces. Intravenous (iv) injections into a warmed lateral tail vein, ip injections, and euthanasia were performed under isoflurane anesthesia. Treatment dose volumes were adjusted based on the weight of the mouse, with a 0.5 mL volume corresponding to a 35-g mouse. To probe the effect of delayed treatment, groups of four mice were injected iv with a single dose of ^153^Gd-citrate, and ligand or control saline solutions were administered ip once at the following post-contamination treatment times: 1 h, 24 h, 48 h. To probe the effect of prophylactic treatment, groups of four mice were first administered ligand or control saline solutions ip once at the following pre-contamination treatment times: −1 h and −24 h. Mice were then injected iv with a single dose of ^153^Gd-citrate. Excreta were collected daily for 4 days until scheduled necropsy (4 days after metal challenge). Mice were euthanized by cervical dislocation over their respective cage to collect the excreta expelled at death, and immediately frozen for later dissection.

### Tissue Sampling and Processing

Brains, thymuses, hearts, lungs, livers, spleens, and kidneys were dissected, and the abdominal remainder tissue (ART, which includes intact gastrointestinal (GI) tract, reproductive organs, urinary bladder, and abdominal fat) was removed. The organ samples and partially eviscerated carcasses were managed as individual samples. Feces samples were separated manually from urine-stained cellulose bedding and treated as group samples (one group per cage). All samples were dried at 100 °C and dry-ashed at 575 °C. The ashed samples were treated with concentrated HNO_3_. These acidified solutions were then homogenized in dilute HNO_3_ and mixed with Ultima Gold (Perkin Elmer, Shelton, CT, USA) for detection by liquid scintillation counting (Packard Tri-Carb model B4430, Perkin Elmer). Additional details of autopsy procedures, tissue and excreta collection and processing, as well as radioactivity measurements and methods of data reduction have been previously reported for similar experiments with several other long-lived radiotracers^10,33^.

### Data management and analysis

All experiments used radioactive ^153^Gd as a contaminant and were managed as metabolic balance studies, in which all tissues and excreta were radioanalyzed; average radiochemical recoveries were all greater than 90% injected dose. All manipulation of the raw data was performed using the R program. The experimental data are reported as radionuclide fractions, expressed as percent of recovered ^153^Gd (%RD), and both graphical and tabulated values are reported as arithmetic means ± SD. The statistical significance of changes in ^153^Gd concentration was determined using one-way analysis of variance (ANOVA), and changes both pairwise and relative to the control group were assessed using Tukey’s and Dunnett’s tests respectively, as implemented in the Multcomp package for R. Unless otherwise noted, changes were considered significant for *P* < 0.001. Tests for statistical outliers were performed using Dixon’s *Q* test at a 95% confidence level as implemented in the Outliers package for R, where ${Q}=\frac{{x}_{n}-{x}_{n-1}}{{x}_{n}-{x}_{1}}$. The R script used to perform the analysis, as well as a complete ANOVA analysis, can be found at http://github.com/julianrees/2017_GdDecorporation.

### Speciation calculations

All speciation calculations were performed using *Hyperquad Simulation and Speciation* software (*HySS*) in its high precision mode. *HySS* software was implemented with the published stability constants for the soluble species of Gd^3+^, Zn^2+^, and Ca^2+^ with the synthetic chelators of interest (DOTA, DTPA, DTPA-BMA, EDTA, and HOPO) and the endogenous chelators (hydroxides, phosphates, carbonates, citrates, lactates, and oxalates). The protonation constants of the chelators were also implemented in *HySS* software. Each speciation simulation contained between 40 and 74 equilibria. The stability constants used in this study are summarized in Table S3.

## Electronic supplementary material

Supplementary Information
