# Supplementary material for: Evaluating the potential of chelation therapy to prevent and treat gadolinium deposition from MRI contrast agents
Source: Sci Rep. 2018 Mar 13;8:4419. doi: 10.1038/s41598-018-22511-6 (PMC5849765; doi:10.1038/s41598-018-22511-6)

Supporting Information

Evaluating the potential of chelation therapy to prevent and treat gadolinium deposition from MRI contrast agents

Julian A. Rees[a], Gauthier J.-P. Deblonde[a], Dahlia D. An[a], Camille Ansoborlo[a], Stacey S. Gauny[a], Rebecca J. Abergel[a,b,*]

[a]Chemical Sciences Division, Lawrence Berkeley National Laboratory, 1 Cyclotron Road, Berkeley, CA 94720, USA

[b]Nuclear Engineering Department, University of California, Berkeley, CA 94720, USA

*To whom correspondence should be addressed. E-mail: rjabergel@lbl.gov; Fax: +1 510 486 5596; Tel: +1 510 486 5249.

Supporting Table S1. Treatment details for each group. Treatment times are referenced to the ^{153}Gd contamination event.

Group	Ligand	Time	Treatment
A	Saline	1 hr pre	100 μmol / kg
B	HOPO	24 hr pre	100 μmol / kg
C	HOPO	1 hr pre	100 μmol / kg
D	DTPA	1 hr pre	100 μmol / kg
E	HOPO	1 hr post	100 μmol / kg
F	DTPA	1 hr post	100 μmol / kg
G	HOPO	24 hr post	100 μmol / kg
H	HOPO	48 hr post	100 μmol / kg

Supporting Table S2. ^{153}Gd content of murine tissues and excreta for all groups. Data are reported as arithmetic means ± standard deviations in % of the recovered dose. Excreta were collected by group, therefore only the mean is reported.

Table 1: ^{153}Gd con
deviations in % of t

Group	Skeleton
A	40.5±1.3
B	3.88±0.99
C	0.0359±0.072
D	7.59±1
E	13.6±1.6
F	31.4±1.7
G	29.2±1.2
H	28.4±1

Supporting Table S3. Equilibria and corresponding stability constants used in the speciation study.

**Equilibrium**	**log K**	**Reference**
$Gd^{3+} + PO_4^{3-} = [GdPO_4]_{(aq)}$	12.19	NIST
$Gd^{3+} + HPO_4^{2-} = [GdHPO_4]^+$	5.91	NIST
$Gd^{3+} + 2\ HPO_4^{2-} = [Gd(HPO_4)_2]^-$	9.97	NIST
$Gd^{3+} + H_2PO_4^- = [GdH_2PO_4]^{2+}$	2.74	NIST
$Gd^{3+} + C_2O_4^{2-} = [GdC_2O_4]^+$	4.77	NIST
$Gd^{3+} + 2\ C_2O_4^{2-} = [Gd(C_2O_4)]^-$	8.66	NIST
Gd^{3+} + Lactate$^-$ = [GdLactate]$^{2+}$	2.91	NIST
Gd^{3+} + 2 Lactate$^-$ = [GdLactate$_2$]$^+$	5.04	NIST
Gd^{3+} + 3 Lactate$^-$ = [GdLactate$_3$]	6.24	NIST
Gd^{3+} + Citrate^{3-} + H^+ = [GdHCitrate]	21.2	Heller 2012[a]
Gd^{3+} + 2 Citrate^{3-} + 3 H^+ = [Gd(H_2Citrate)(HCitrate)]$^{2-}$	43.6	Heller 2012[a]
Gd^{3+} + 2 Citrate^{3-} + 2 H^+ = [Gd(HCitrate)$_2$]$^{3-}$	38.5	Heller 2012[a]
Gd^{3+} + 2 Citrate^{3-} = [GdCitrate$_2$]$^{5-}$	21.0	Heller 2012[a]
$Gd^{3+} + CO_3^{2-} = [GdCO_3]^+$	7.64	NIST
$Gd^{3+} + 2\ CO_3^{2-} = [Gd(CO_3)_2]^-$	13.04	NIST
$Gd^{3+} + HCO_3^- = [GdHCO_3]^{2+}$	1.9	NIST
Gd^{3+} + DTPA^{5-} = [GdDTPA]$^{2-}$	22.39	NIST
[GdDTPA]$^{2-}$ + H^+ = [GdHDTPA]$^-$	2.39	NIST
Gd^{3+} + DOTA^{4-} = [GdDOTA]$^-$	24.0	NIST
Gd^{3+} + DTPA-BMA^{3-} = [GdDTPA-BMA]	16.86	NIST
Gd^{3+} + EDTA^{4-} = [GdEDTA]$^-$	17.35	NIST
[GdEDTA]$^-$ + H^+ = [GdHEDTA]	1.3	NIST
Gd^{3+} + HOPO^{4-} = [GdHOPO]$^-$	20.5	Sturzbecher-Hoehne 2011[b]
[GdHOPO]$^-$ + H^+ = [GdHHOPO]	1.2	Sturzbecher-Hoehne 2011[b]
$Gd^{3+} + H_2O = [GdOH]^{2+} + H^+$	-8.1	Sturzbecher-Hoehne 2011[b]
$Gd^{3+} + 2\ H_2O = [Gd(OH)_2]^+ + 2\ H^+$	-14.5	Sturzbecher-Hoehne 2011[b]
$Gd^{3+} + 3\ H_2O = [Gd(OH)_3] + 3\ H^+$	-24.1	Sturzbecher-Hoehne 2011[b]
Zn^{2+} + DTPA^{5-} = [ZnDTPA]$^{3-}$	18.2	NIST
[ZnDTPA]$^{3-}$ + H^+ = [ZnHDTPA]$^{2-}$	5.6	NIST
[ZnDTPA]$^{3-}$ + Zn^{2+} = [Zn_2DTPA]$^-$	4.48	NIST
Zn^{2+} + EDTA^{4-} = [ZnEDTA]$^{2-}$	16.5	NIST
[ZnEDTA]$^{2-}$ + H^+ = [ZnHEDTA]$^-$	3.0	NIST
Zn^{2+} + DTPA-BMA^{3-} = [ZnDTPA-BMA]$^-$	12.04	NIST
$Zn^{2+} + HPO_4^{2-} = [ZnHPO_4]$	2.46	NIST
$Zn^{2+} + H_2PO_4^- = [ZnH_2PO_4]^+$	1.2	NIST
Zn^{2+} + Oxalate^{2-} = [ZnOxalate]	4.0	NIST
Zn^{2+} + 2 Oxalate^{2-} = [ZnOxalate$_2$]$^{2-}$	6.45	NIST
Zn^{2+} + Lactate$^-$ = [ZnLactate]$^+$	1.86	NIST
Zn^{2+} + 2 Lactate$^-$ = [ZnLactate$_2$]	2.6	NIST
Zn^{2+} + 3 Lactate$^-$ = [ZnLactate$_3$]$^-$	3.4	NIST
$Zn^{2+} + CO_3^{2-} = [ZnCO_3]$	3.9	NIST
$Zn^{2+} + 2\ CO_3^{2-} = [Zn(CO_3)_2]^{2-}$	7.3	NIST

$Zn^{2+} + HCO_3^- = [ZnHCO_3]^+$	1.5	NIST
Zn^{2+} + Citrate^{3-} = [ZnCitrate]$^-$	4.93	NIST
Zn^{2+} + 2 Citrate^{3-} = [ZnCitrate$_2$]$^{4-}$	6.8	NIST
Zn^{2+} + HCitrate^{2-} = [ZnHCitrate]	3.00	NIST
Zn^{2+} + H$_2$Citrate$^-$ = [ZnH$_2$Citrate]$^+$	1.2	NIST
$Zn^{2+} + HO^- = [ZnOH]^+$	4.6	NIST
$Zn^{2+} + 2\ HO^- = [Zn(OH)_2]$	11.1	NIST
$Zn^{2+} + 3\ HO^- = [Zn(OH)_3]^-$	13.6	NIST
$Zn^{2+} + 4\ HO^- = [Zn(OH)_4]^{2-}$	14.8	NIST
Ca^{2+} + DTPA^{5-} = [CaDTPA]$^{3-}$	10.75	NIST
[CaDTPA]$^{3-}$ + H^+ = [CaHDTPA]$^{2-}$	6.11	NIST
[CaDTPA]$^{3-}$ + Ca^{2+} = [Ca$_2$DTPA]$^-$	1.6	NIST
Ca^{2+} + EDTA^{4-} = [CaEDTA]$^{2-}$	10.65	NIST
Ca^{2+} + DTPA-BMA^{3-} = [CaDTPA-BMA]$^-$	7.17	NIST
$Ca^{2+} + HPO_4^{2-} = [CaHPO_4]$	1.62	NIST
$Ca^{2+} + H_2PO_4^- = [CaH_2PO_4]^+$	0.6	NIST
Ca^{2+} + Oxalate^{2-} = [CaOxalate]	2.46	NIST
$Ca^{2+} + CO_3^{2-} = [CaCO_3]$	3.22	NIST
$Ca^{2+} + HCO_3^- = [CaHCO_3]^+$	0.29	NIST
Ca^{2+} + Citrate^{3-} = [CaCitrate]$^-$	3.48	NIST
Ca^{2+} + HCitrate^{2-} = [CaHCitrate]	2.07	NIST
Ca^{2+} + Lactate$^-$ = [CaLactate]$^+$	1.12	NIST
Ca^{2+} + 2 Lactate$^-$ = [CaLactate$_2$]	1.62	NIST
$Ca^{2+} + HO^- = [CaOH]^+$	1.3	NIST
$PO_4^{3-} + H^+ = HPO_4^{2-}$	11.8	NIST
$HPO_4^{2-} + H^+ = H_2PO_4^-$	6.88	NIST
$H_2PO_4^- + H^+ = = H_3PO_4$	1.99	NIST
$CO_3^{2-} + H^+ = HCO_3^-$	9.9	NIST
$HCO_3^- + H^+ = H_2CO_3$	6.13	NIST
Oxalate^{2-} + H^+ = HOxalate$^-$	3.82	NIST
HOxalate$^-$ + H^+ = H$_2$Oxalate	1.2	NIST
Lactate$^-$ + H^+ = HLactate	3.67	NIST
Citrate-O$^-$ + H^+ = Citrate^{3-} (Hydroxyl proton)	13.5	Heller 2012[a]
Citrate^{3-} + H^+ = HCitrate^{2-}	5.7	Heller 2012[a]
HCitrate^{2-} + H^+ = H$_2$Citrate$^-$	4.4	Heller 2012[a]
H$_2$Citrate$^-$+ H^+ = H$_3$Citrate	2.9	Heller 2012[a]
DOTA^{4-} + H^+ = HDOTA^{3-}	11.2	NIST
HDOTA^{3-} + H^+ = H$_2$DOTA^{2-}	9.73	NIST
H$_2$DOTA^{2-} + H^+ = H$_3$DOTA$^-$	4.44	NIST
H$_3$DOTA$^-$ + H^+ = H$_4$DOTA	4.34	NIST
H$_4$DOTA + H^+ = H$_5$DOTA$^+$	2.35	NIST
DTPA^{5-} + H^+ = HDTPA^{4-}	10.4	NIST
HDTPA^{4-} + H^+ = H$_2$DTPA^{3-}	8.55	NIST
H$_2$DTPA^{3-} + H^+ = H$_3$DTPA^{2-}	4.28	NIST

$H_3DTPA^{2-} + H^+ = H_4DTPA^-$	2.7	NIST
$H_4DTPA^- + H^+ = H_5DTPA$	2.0	NIST
$H_5DTPA + H^+ = H_6DTPA^+$	1.6	NIST
$H_6DTPA^+ + H^+ = H_7DTPA^2$	0.7	NIST
$DTPA\text{-}BMA^{3-} + H^+ = HDTPA\text{-}BMA^{2-}$	9.37	NIST
$HDTPA\text{-}BMA^{2-} + H^+ = H_2DTPA\text{-}BMA^-$	4.38	NIST
$H_2DTPA\text{-}BMA^- + H^+ = H_3DTPA\text{-}BMA$	3.31	NIST
$HOPO^{4-} + H^+ = HHOPO^{3-}$	6.64	Abergel 2009[c]
$HHOPO^{3-} + H^+ = H_2HOPO^{2-}$	5.68	Abergel 2009[c]
$H_2HOPO^{2-} + H^+ = H_3HOPO^-$	5.01	Abergel 2009[c]
$H_3HOPO^- + H^+ = H_4HOPO$	3.87	Abergel 2009[c]
$H_2O = H^+ + HO^-$	-13.77	NIST

Notes: Most log K values are given for an ionic strength of 0.1M and at 25°C, see corresponding reference for more information. NIST: A. E. Martell, R. M. Smith, R. J. Motekaitis, NIST Critically Selected Stability Constants of Metal Complexes: Version 8.0. a: Heller et al. *Dalton Trans.*, 2012, 41, 13969. Values for the Gd-citrate complexes were considered identical to those of the Eu-citrate complexes. b: Sturzbecher-Hoehne et al., *Dalton Trans.*, 2011, 40, 8340-8346. The protonation constant of the $[GdHOPO]^-$ complex was considered similar to that of the $[EuHOPO]^-$ complex. c: Abergel et al. *Inorg. Chem*. 2009, 48, 10868–10870.

Supporting Figure S1. Murine organ content of ^{153}Gd four days following contamination via intravenous injection into a warmed lateral tail vein, reported as % RD for each treatment group. Treatments were administered at a dose of 100 μmol / kg via ip injection at times varying from 24 h pre- to 48 h post-Gd injection. Data points correspond to the values obtained for each mouse in a group (n = 4). Organs are ranked in order of contribution to the total RD. Control = squares, HOPO = triangles, DTPA = circles.

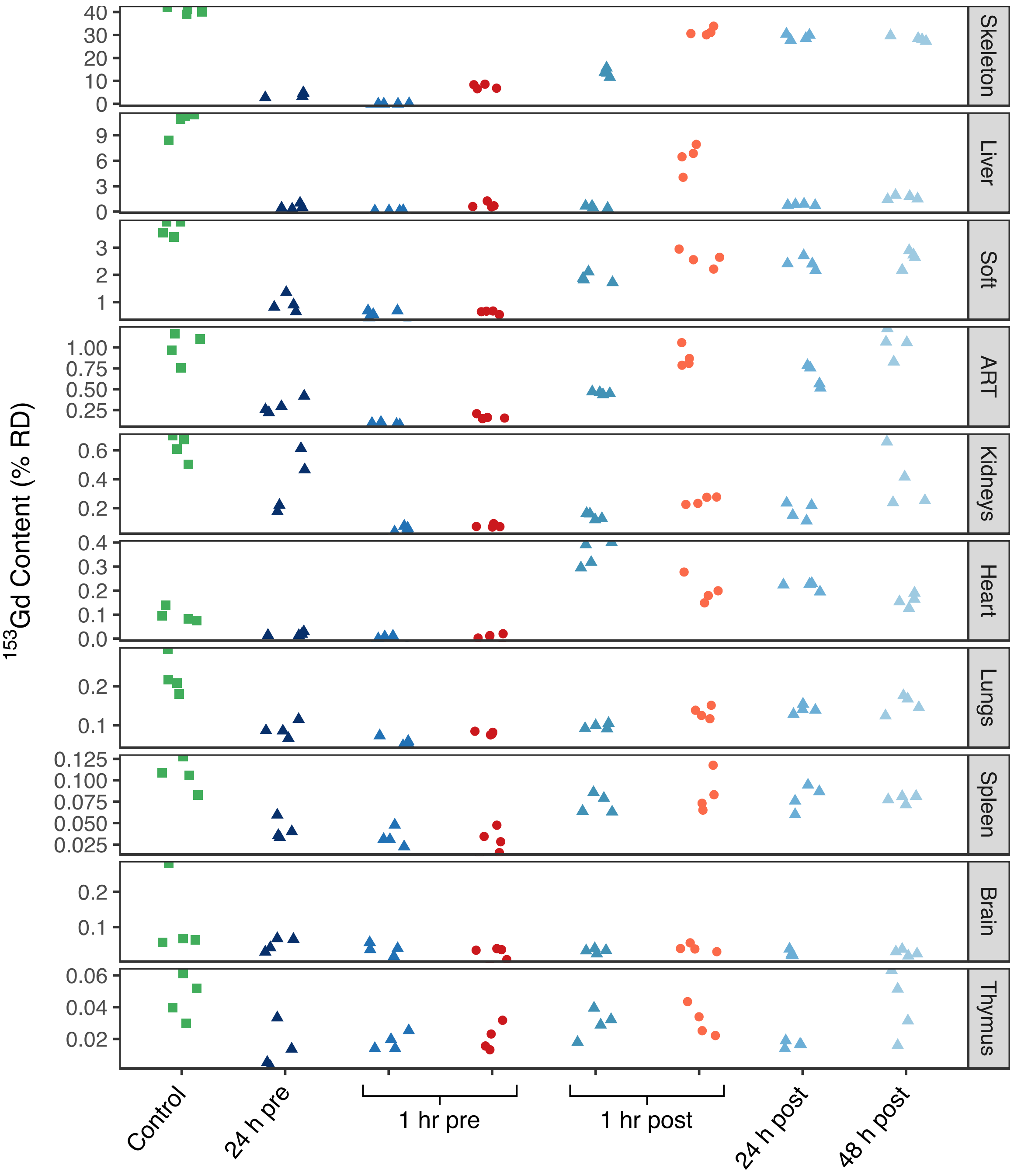

Supporting Figure S2. Cumulative ^{153}Gd excretion by study day and excretion route, reported as percentage of the total recovered dose. Treatments were administered at a dose of 100 μmol / kg via ip injection at times varying from 24 h pre- to 48 h post-Gd injection. Consistent with ^{153}Gd content of body, pre-treatment with HOPO shows it is most efficacious.

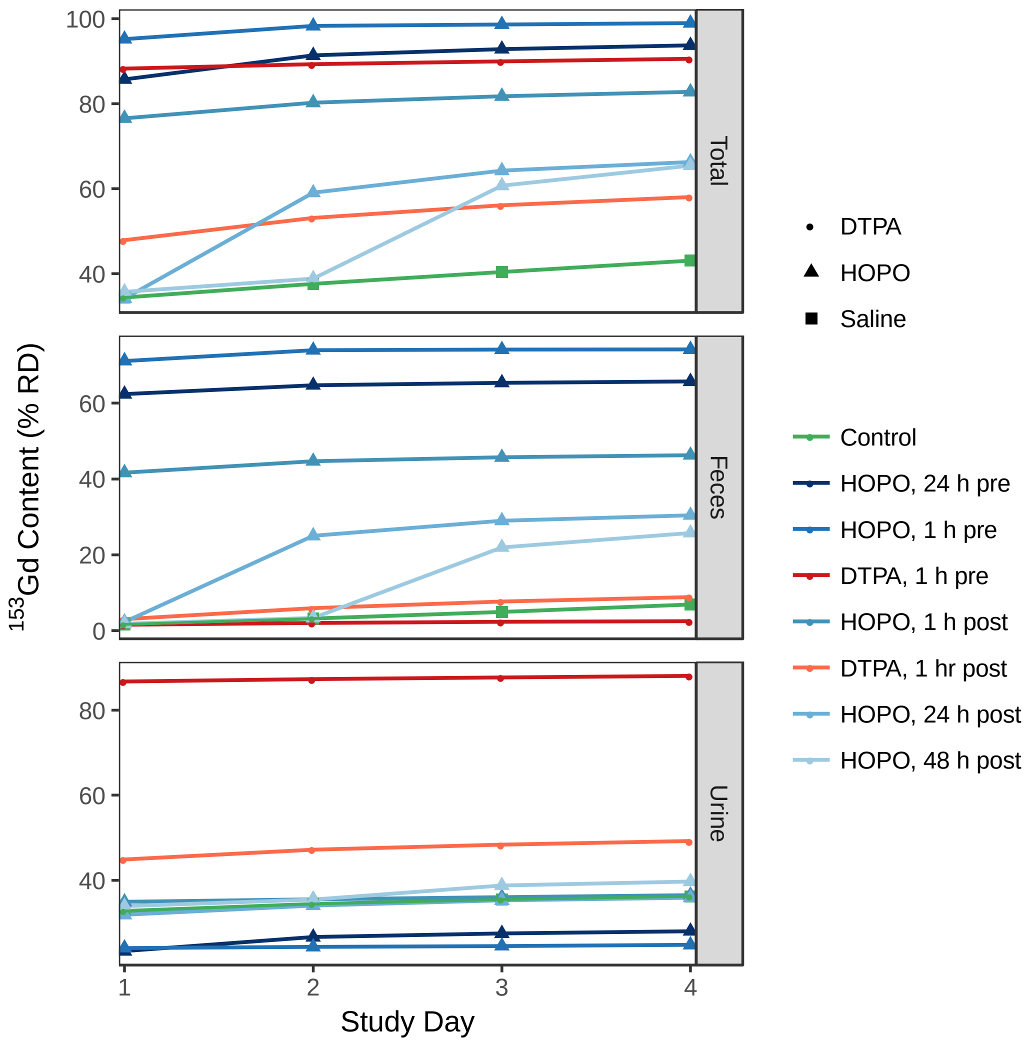

Supporting Figure S3. Calculated speciation of gadolinium under physiological conditions and for a concentration of Gd corresponding to 5% of the typical amount of GBCA injected to a patient. [Gd] = 7.15 μM. [Phosphate] = 1.1 mM, [Carbonate] = 25 mM, [Oxalate] = 9.2 μM, [lactate] = 1.5 mM, [Citrate] = 160 μM. pH = 7.4. Citrate species are displayed in yellow, the phosphate species in red, and the carbonate species in blue.

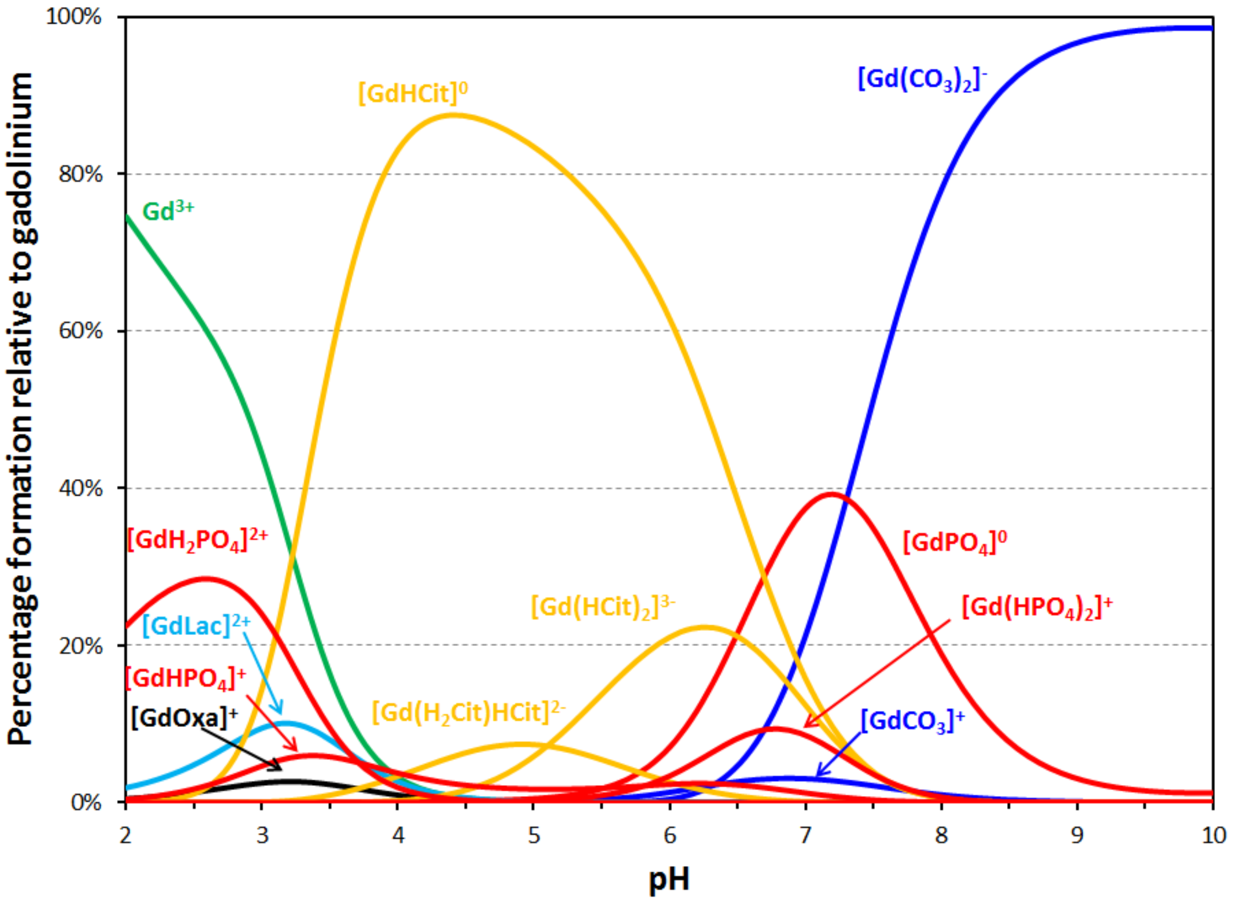

Supporting Figure S4. Calculated speciation of gadolinium under physiological conditions and for a concentration of Gd corresponding to 0.05% of the typical amount of GBCA injected to a patient. [Gd] = 715 nM. [Phosphate] = 1.1 mM, [Carbonate] = 25 mM, [Oxalate] = 9.2 µM, [lactate] = 1.5 mM, [Citrate] = 160 µM. pH = 7.4. Citrate species are displayed in yellow, the phosphate species in red, and the carbonate species in blue.

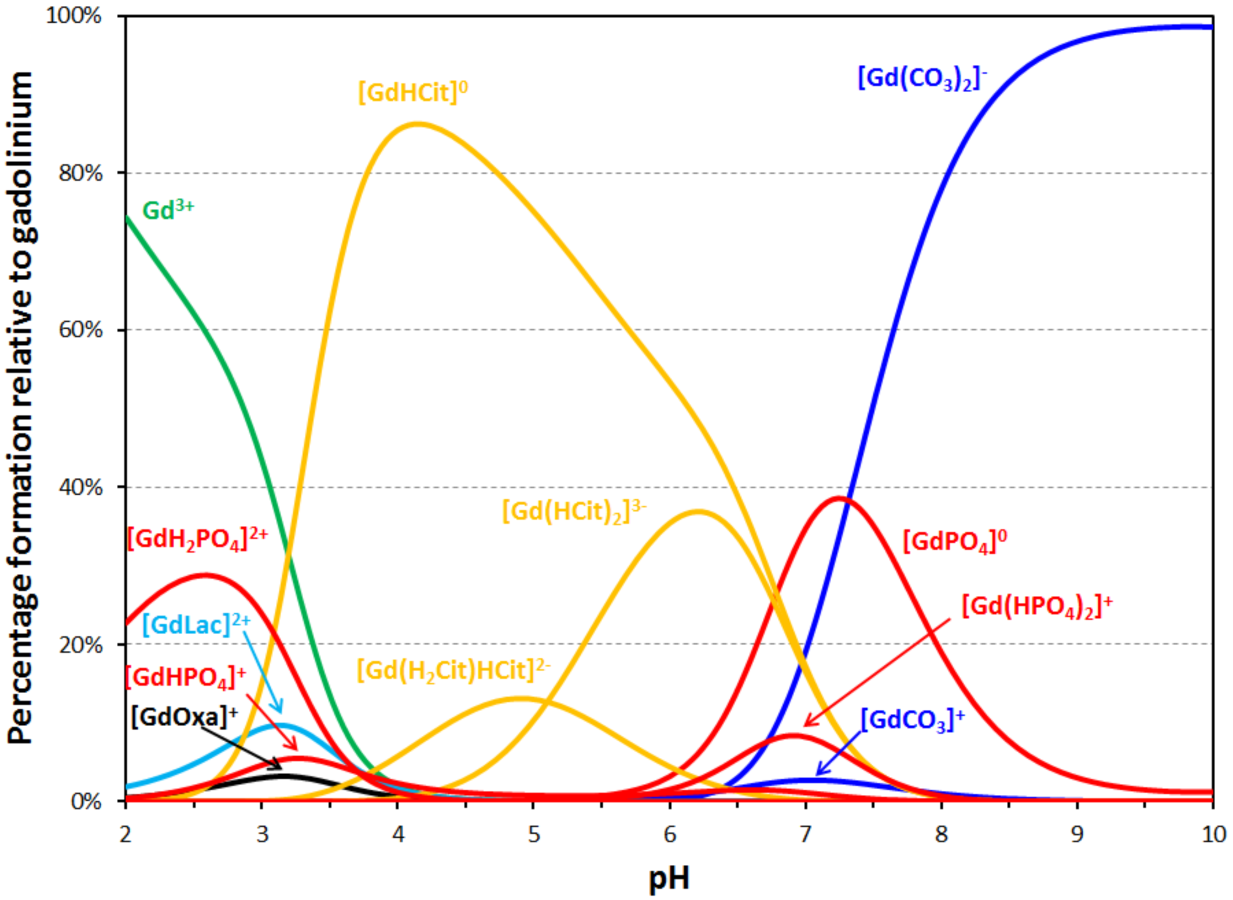

Supporting Figure S5. Calculated percentage of gadolinium bound to DTPA as a function of dilution and in the presence of bio-relevant chelators. Calculations in the presence of calcium (1.1 mM) or zinc (15 μM) ions are also given for comparison. Total concentrations of phosphates (1.1 mM), carbonates (25 mM), oxalates (9.2 μM), lactates (1.5 mM), and citrates (160 μM) held constant to match physiological conditions. Ratio Gd/DTPA = 1.0 mol/mol. pH = 7.4. See Table S3 for stability constants used in these speciation simulations.

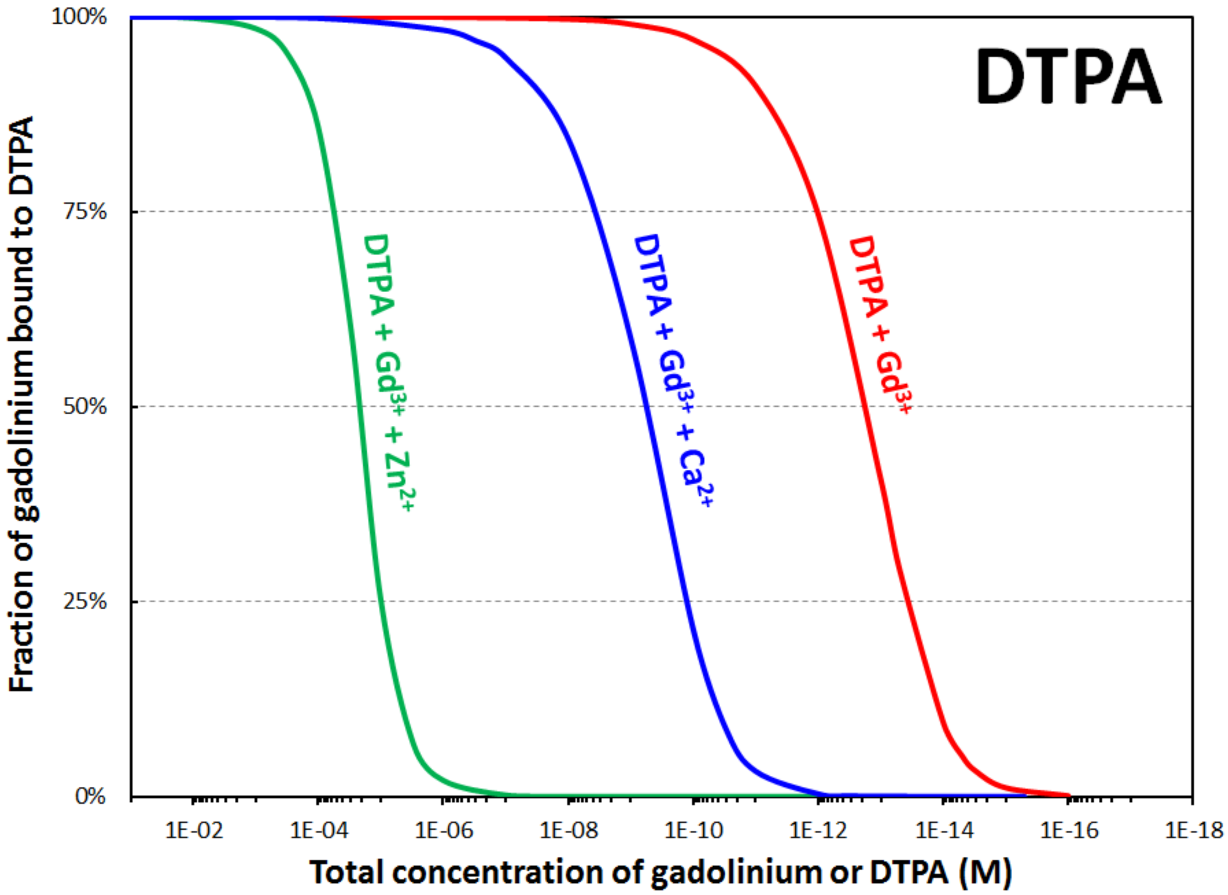

Supporting Figure S6. Calculated percentage of gadolinium bound to DTPA-BMA as a function of dilution and in the presence of bio-relevant chelators. Calculations in the presence of calcium (1.1 mM) or zinc (15 μM) ions are also given for comparison. Total concentrations of phosphates (1.1 mM), carbonates (25 mM), oxalates (9.2 μM), lactates (1.5 mM), and citrates (160 μM) held constant to match physiological conditions. Ratio Gd/DTPA-BMA = 1.0 mol/mol. pH = 7.4. See Table S3 for stability constants used in these speciation simulations.

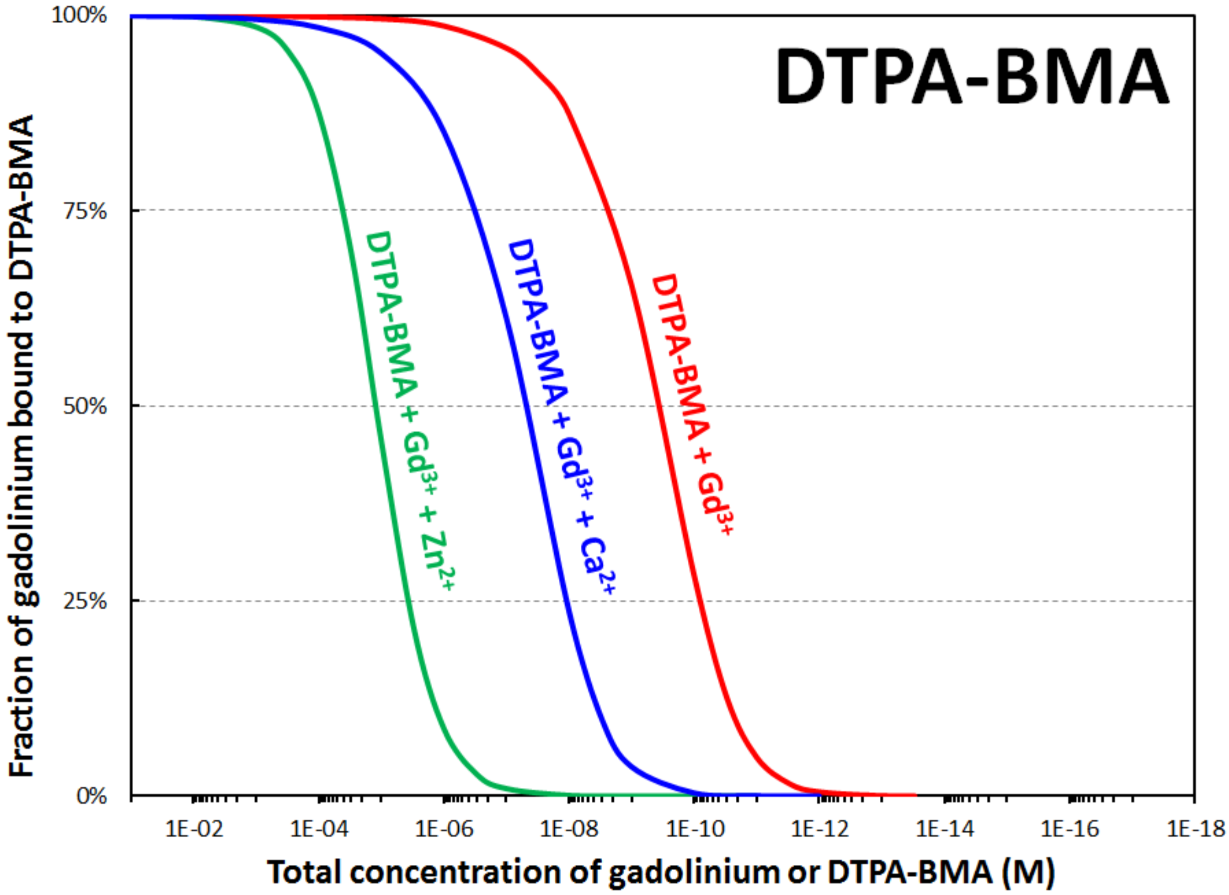

Supplement: Supplementary file 1 — Supplementary Information [file 41598_2018_22511_MOESM1_ESM.pdf]
